# Scalable efficient expansion of mesenchymal stem cells in xeno free media using commercially available reagents

**DOI:** 10.1186/s12967-015-0561-6

**Published:** 2015-07-17

**Authors:** Neil H Riordan, Marialaura Madrigal, Jason Reneau, Kathya de Cupeiro, Natalia Jiménez, Sergio Ruiz, Nelsy Sanchez, Thomas E Ichim, Francisco Silva, Amit N Patel

**Affiliations:** Medistem Panama, Inc., Building 221, City of Knowledge, Clayton, Panama City, Republic of Panama; Indicasat AIP Panama, City of Knowledge, Rep. of Panama; Acharya Nagarjuna University, Guntur, India; Amniotic Therapies, LLC, Farmers Branch, TX USA; Coma Research Institute, La Jolla, CA USA; University of Utah School of Medicine, Salt Lake City, UT USA

## Abstract

**Background:**

The rapid clinical translation of mesenchymal stem cells (MSC) has resulted in the development of cell-based strategies for multiple indications. Unfortunately one major barrier to widespread implementation of MSC-based therapies is the limited supply of fetal calf serum (FCS) used to expand cells to therapeutic numbers. Additionally, the xenogeneic element of fetal calf serum has been previously demonstrated to stimulate antibody mediated reactions and in some cases sensitization leading to anaphylaxis.

**Method:**

XcytePLUS™ media, a human platelet lysate based product, was used to supplement the culture medium at 5, 7.5 and 10% and compared to fetal calf serum at 10%, for human umbilical cord MSC expansion. Properties of the expanded cells were investigated.

**Results:**

This study demonstrated equivalent or superior effects of human platelet lysate compared to standard FCS supplemented media, based on doubling rate, without loss of identity or function, as demonstrated with flow cytometry characterization. Differentiation into osteocytes, adipocytes and chondrocytes was comparable from cells expanded in either media supplement.

**Conclusions:**

These data support the implementation of human platelet lysate supplemented media as an alternative to xenogeneic containing preparations which may lead to safer MSC products with therapeutic uses.

## Background

Tissue culture originated with the work of Alexis Carrell at the beginning of the 20th century utilizing a variety of undefined media additives such as embryonic and muscle extracts mixed with saline plasma, which were able to maintain cardiac cells in vitro and allow for contractile function [1]. The introduction of fetal calf serum (FCS) as a media additive revolutionized the field of tissue culture and allowed for laboratories to maintain stable cell lines in vitro, which catalyzed major efforts in cancer research [2]. The concept of utilizing cells as therapeutics originated in the bone marrow transplant area, in which uncultured donor bone marrow stem cells were used for hematopoietic support to allow for high dose chemotherapy and radiation therapy [3]. The utilization of cellular immunotherapy, in the 1980s was the first major clinical experiments that required expansion of cells in vitro. In these early experiments, it was demonstrated that fetal calf serum posed some potential to induce adverse effects. For example, a study reported that syngeneic lymphocytes cultured in FCS and subsequently administered to HIV patients resulted in Arthus reaction and fever in recipients, which was associated with antibodies to FCS components [4]. Other studies have shown that both pre-existing natural, as well as inducible antibodies are present in patients that recognize various components of FCS [5–8]. Additionally, activation of both T cell and NK cell responses have been reported subsequent to stimulation with FCS [9, 10].

Mesenchymal stem cells (MSC) are fibroblast-shaped cells capable of multigenic differentiation into bone, fat and cartilage, as well as differentiation into neuronal, hepatic and pancreatic tissue [11]. A number of clinical trials have been performed utilizing MSC in conditions ranging from hepatic failure [12], type 1 diabetes [13], and stroke [14]. The majority of these clinical trials have utilized MSCs that are grown in FCS containing media. Overall safety has been demonstrated of MSC, as reported in a meta-analysis of clinical trials containing over 1,000 patients [15]. Interestingly, in these trials administration doses were 1–3 injections, thus substantially decreasing the possibility of sensitization. Despite this, some evidence of sensitization has been reported. Specifically, Le Blanc et al. demonstrated in 12 patients being administered bone marrow MSC for treatment of steroid resistant graft versus host disease (GVHD) the development of antibodies to fetal calf serum proteins as detected by ex vivo treatment of fetal calf proteins [16].

In order to overcome limitations associated with FCS, numerous groups have developed serum-free tissue culture media compositions for growing and expansion of MSC. Platelet derived growth factor (PDGF) is a potent cellular mitogen and has been reported to be a significant component of FCS allowing for cellular proliferation in vitro [17]. Since platelet lysate contains significant concentrations of PDGF, as well as numerous other mitogenic factors [18], investigators have utilized this as a potential substitute for FCS. In 2006 Müller et al. reported that bone marrow MSC cultures can be initiated and maintained in media in which FCS was substituted with platelet lysate [19]. The MSC grown in platelet lysate substituted media retained both differentiation, as well as immune modulatory activity. Numerous other studies have demonstrated that various platelet lysate preparations are capable of maintaining or increasing proliferation of bone marrow MSC as a substitute for FCS [20–35]. Another report on 213 patients treated with autologous BM MSC cultured in platelet lysate reported no adverse reactions subsequent to intradisc and intrajoint administration [36]. Some characterization has occurred of active components of platelet lysate. Specifically, antibody blocking studies have shown that up to 75% inhibition of MSC proliferation in response to platelet lysate was achieved with a combination of anti-bFGF + anti-PDGF-BB and anti-bFGF + anti-TGF-β1 + anti-PDGF-BB. Interestingly, various combinations of recombinant PDGF-BB, bFGF and TGF-β1 were not sufficient to promote cell proliferation, implying some components still remain unknown [37].

Despite advancements that have been reported utilizing platelet lysate, several obstacles still remain for large-scale commercial application, namely: (a) establishing the role that platelet lysate plays in increasing anti-inflammatory activity of MSC when compared to culture in FCS [38, 39]; (b) significant variation in MSC cultures depending on platelet donor profiles [40], with age being a contributing factor [41]; and (c) institution-specific proprietary methods for large-scale commercial production of platelet lysate, and the roles of factors such as heparin [42], or fibrinogen [39], concentration post large-scale production. Addressing these points will allow for a better standardized method for large-scale commercial production.

Here we present data using a commercially available platelet-lysate based media, XcytePLUS™, to culture human umbilical cord’s Wharton’s Jelly MSC (WJ-MSC) in comparison to media containing FCS. Additionally, the culture system described is completely free of xenogeneic components in that dissociation media did not contain bovine trypsin. We report equivalent or superior proliferation, and differentiation activity of WJ-MSC cultured in XcytePLUS™ media as compared to FCS.

## Methods

### Cells and tissue culture

Wharton’s Jelly mesenchymal stem cells were isolated from healthy donors according to Secco et al. [43]. Briefly, umbilical cords of minimum 25 cm long were dissected, and cut in pieces of 8 cm, and they were digested using Collagenase 1.67% (Sigma C9891) during 60 min at 37°C. Tissue was washed twice and all supernatant was centrifuged to collect the pellet and then plated at 1 × 10^4^ cells/cm^2^. Cells were cultured using MEM alpha (Life 32561102) supplemented with 2 mM GlutaMax (Life 35050-079) and 10% FCS (Life 16000044).

All Isolated MSCs were frozen in Passage 2. Immunophenotype characterization was performed and based on those results, four lots were selected from a stock of cells to continue the experiments.

MSCs were thawed and plated into 6 well plates (6 wells per treatment), 5 × 10^4^ cells/well, using XcytePLUS™ (iBiologics XPGS-001-500) (5, 7.5, and 10%) or 10% FCS supplemented media in two different laboratories. Cells were grown up to 80–90% confluence, then passaged using Tryple Express (Life Technologies), splits were 1:6, up to passage 6. Cell counts were performed every passage before re-plating to determine cell-doubling time. Finally, cells were frozen for further analysis of membrane markers expression and differentiation.

### Flow cytometry

Flow cytometry was performed using Guava EasyCyte Mini (Millipore) flow cytometer. Antibodies CD105, CD73, CD90, CD34, CD45 were purchased from (BD pharmingen), isotype controls for FITC and RPE were utilized.

### Doubling rate

Doubling rate of the cells was assessed by counting the cells in every passage. Number of doublings was calculated by the equation:

$$ {\text{X}} = \left( {{ \log }\left( {\text{n}} \right) \, - { \log }\left( {\text{n}}_0 \right)} \right)/{ \log }\left( 2\right), $$

n = number of cells obtained, n_0_ = number of cells plated.

### Statistical analysis

For the statistical analysis, comparison of averages were made using GraphPad (Prism 6) software. Two-way ANOVA with Tukey test was used for doublings per day, cumulative number of cells and characterization analysis. One-way ANOVA was used for inter-laboratory analysis. In all tests a confidence interval of 95% was used.

### Differentiation assays

Two lots were used for the differentiation assay. Briefly, 24 well plates were used and differentiation medium was prepared using DMEM low glucose, without phenol red (Life Technologies) supplemented with 2 mM glutaMax and 10% FCS. Adipogenesis media was supplemented with 1 μM dexamethasone, 500 μM 3-isobutyl-1-methylxantine, 60 μM indometacine, 5 μg/ml insulin. Chondrogenesis media with 0.1 μM dexamethasone, 50 μM ascorbate-2-phosphate, 1 mM sodium pyruvate, 1% ITS Premix, 10 ng/ml TGF-β1, and Osteogenesis media with 0.1 μM dexamethasone, 50 μM ascorbate-2-phosphate, 10 mM β-glycerophosphate. After 21 days, alcian blue, alizarin red and oil red O were used to stain chondrocytes, osteocytes and adipocytes, respectively.

## Results

### Doubling rate superior with WJ-MSC cultured in XCyte Media versus FCS

**Figure 1:**
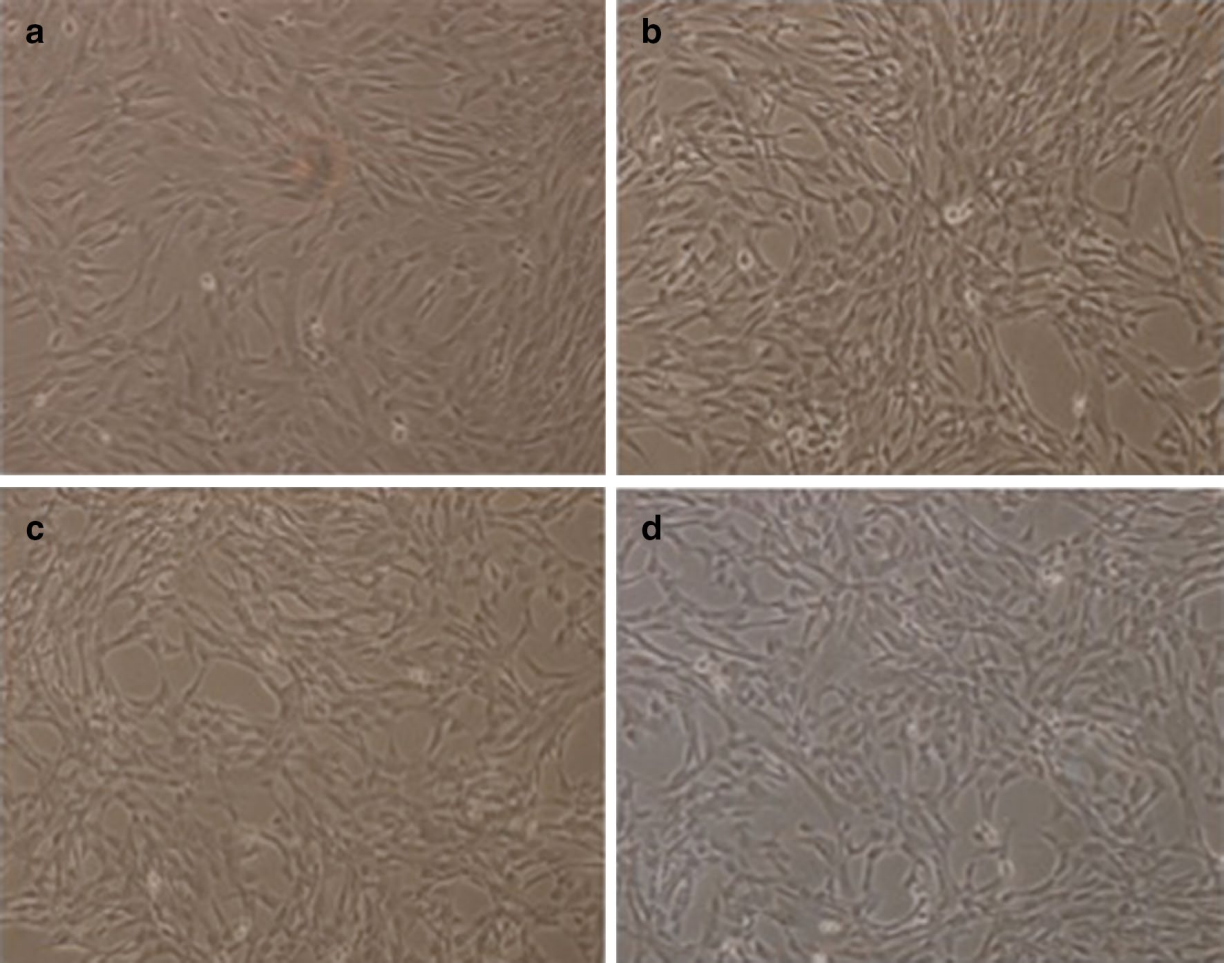
Morphology of WJ-MSC is not altered by supplementation of culture media with XcytePLUS™ compared to Fetal Calf Serum (FCS). 10X inverted phase contrast microscope images of WJ-MSC cultured in **a** 10% FCS, **b** 5% XcytePLUS™, **c** 7.5% XcytePLUS™ and **d** 10% XcytePLUS™ on passage 5.

**Figure 2:**
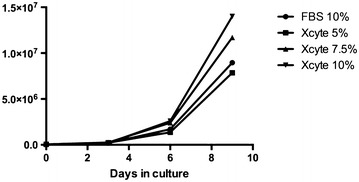
Superior expansion of WJ-MSC by culture media supplemented with XcytePLUS™ compared to Fetal Bovine/Calf Serum (FBS). Passage 5 WJ-MSC were cultured in media supplemented with the indicated concentrations of XcytePLUS™ or FBS. Cell quantification was performed by manual counting as described in “Methods”. The average represent four different lots run in two different laboratories.

**Figure 3:**
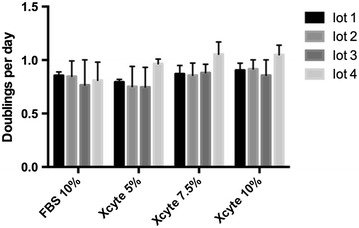
WJ-MSC doubling time similar between different lots. Passage 5 WJ-MSC were cultured in media supplemented with the indicated concentrations of XcytePLUS™ or Fetal Bovine/Calf Serum (FBS). Cell quantification was performed by manual counting, and doubling time was calculated as described in “Methods”. Average doublings per day for four lots using FBS, and different XcytePLUS™ concentrations, in two laboratories.

**Figure 4:**
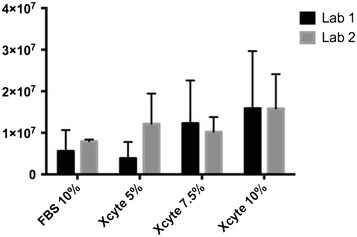
Inter-laboratory comparison of WJ-MSC expansion. Passage 6 cells were cultured in media supplemented with the indicated concentrations of XcytePLUS™ or Fetal Bovine/Calf Serum (FBS). Cell quantification was performed by manual counting as described in “Methods”.

WJ-MSC after 5 passages were plated in media containing 5, 7.5 and 10% XcytePLUS™, or 10% FCS supplemented media and cultured for 9 days. Morphological differences were not found between the treatment groups (Figure 1). Significantly higher number of WJ-MSC were collected on day 9 with the 10% XcytePLUS™ supplemented media (average 1.4 × 10^7^) as compared to 10% FCS media (average 7.5 × 10^6^) (p < 0.05). Average numbers are represented in Figure 2. Furthermore, population doublings in 9 days were 8.37 for cells supplemented with 10% XcytePLUS™ and 7.29 using 10% FCS. Doublings per day did not show statistical difference when different lots of XcytePLUS™ were used (Figure 3). Furthermore, doubling rate experiments performed on the same four lots in different laboratories did not show substantial differences (Figure 4).

### Maintenance of MSC phenotype

**Figure 5:**
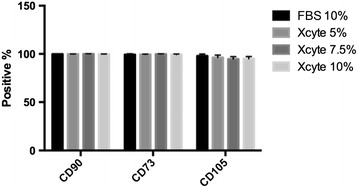
Flow cytometry characterization of XcytePLUS™ versus Fetal Bovine/Calf Serum (FBS) Cultured WJ-MSC. Flow cytometric analysis of indicated MSC markers was performed as described in “Methods”. Marker expression was quantified as percentage positive events detected from gated cells.

In order to assess whether WJ-MSC were not altered by culturing in XcytePLUS™ media from a phenotypic perspective, flow cytometric analysis was performed at the completion of 9 days of culture. As seen in Figure 5, cells maintained the markers of CD105+, CD73+, CD90+ throughout the culture period, which was not affected significantly by the type of media utilized. CD34 and CD45 expression was maintained negative, consistent with classical MSC phenotype.

### Differentiation potential

**Figure 6:**
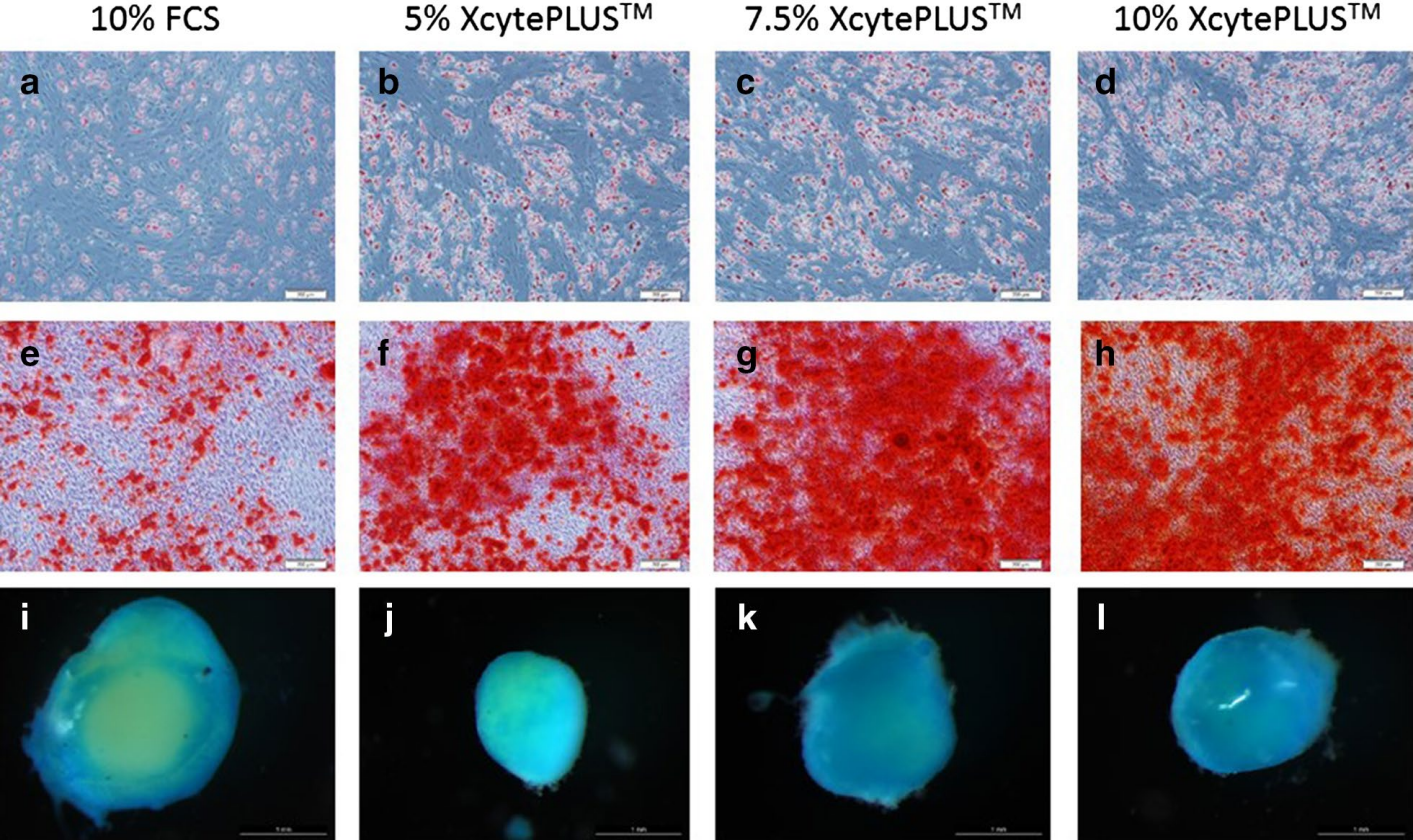
Differentiation potential is retained in culture with XcytePLUS™. WJ-MSC maintained for 6 passages in the indicated culture conditions. Expanded cells were subjected to adipocytic (**a**–**d**), osteogenic (**e**–**h**), and chondrogenic (**i**–**l**) differentiation conditions as described in “Methods”. Morphological observations indicated no differences in differentiation efficacy between the groups.

Cells at passage 6 grown under 5, 7.5 or 10% XcytePLUS™ Media or 10% FCS were exposed to adipogenic, osteogenic and chondrogenic differentiation conditions. As seen in Figure 6, differentiation ability of WJ-MSC was not affected by XcytePLUS™ or FCS supplementation.

## Discussion

The current findings provide for the first time data on reproducibility of a commercially available xenogenic free media supplement and cell dissociation technique assessed across WJ-MSC donors. Equivalent or superior induction of cell proliferation was noted, as well as retention of classical MSC surface markers for 6 passages. Reproducibility of results between two independent laboratories was demonstrated.

Currently there is substantial controversy in the area of platelet lysate based cultures with various investigators providing contradictory results. Furthermore the field is complicated by lack of widespread access to various proprietary reagents utilized in the manufacture of the platelet lysate. Specifically, conditions such as concentration of heparin, fibronectin or donor characteristics all contribute to variability of product. XcytePLUS™ media is commercially available and is based on stringent characterized donors with large lots, thereby decreasing lot to lot variability.

Even though it has been reported that platelet lysate substitution for FCS results in a lower ability to differentiate into both adipogenic and osteogenic lineages, our data does not support this finding [44]. Due to enhanced proliferation rates with platelet lysate media, population doublings could be higher compared to FCS supplemented media. We have demonstrated that population doublings should be controlled so that the cells can retain differentiation ability.

## Conclusions

The large-scale production of clinical grade MSCs demands a xeno-free standardized culture system. Feasibility of the use of XcytePLUS™ was demonstrated with consistent results in proliferation rate, characterization and differentiation potential. XcytePLUS™ is a pooled human platelet lysate product, which allows for a commercial therapeutic MSC product.
